# Best Practices to Overcome Barriers to Capacity Evaluations

**DOI:** 10.1093/geroni/igab046.2597

**Published:** 2021-12-17

**Authors:** Ronan Factora, Saket Saxena

**Affiliations:** Cleveland Clinic, Cleveland, Ohio, United States

## Abstract

Because of the increasing incidence of elder abuse and financial exploitation, Adult Protective Services (APS) cases open for these individuals often relay on capacity evaluations conducted by a clinician to facilitate legal assignment of a surrogate decision maker. Despite this growing need, the number of physicians willing and capable of performing them is limited. Barriers reported by physicians reportedly impair their ability to conduct these evaluations include absence of relevant case information and lack of knowledge about the process itself. Geriatricians and related clinicians often perform these assessments. Sharing best practices with internists and family physicians may help overcome these barriers. A survey of geriatric medicine providers was conducted to identify essential components and questions necessary in the assessment of general decision making capacity. Twenty-nine providers at 6 academic institutions in Ohio responded to the survey and its follow-up inquiries. Though variability existed in evaluation styles and content between providers, a uniform set of recommendations was able to be generated. A total of 13 different summary recommendations were generated from this survey. Necessary components to these evaluations include (1) performance of cognitive testing (2) obtaining collateral information regarding functional status from another trusted individual (3) assessing the individual’s insight into any reported functional impairments or safety concerns by explaining discrepancies between that individual’s own observations and reported concerns from the trusted individual, and (4) using hypothetical situations to assess a person’s judgment and reasoning in addressing any gaps in care or safety concerns raised during the interview.

